# Effects of cancer screening restart strategies after COVID-19 disruption

**DOI:** 10.1038/s41416-021-01261-9

**Published:** 2021-03-15

**Authors:** Lindy M. Kregting, Sylvia Kaljouw, Lucie de Jonge, Erik E. L. Jansen, Elisabeth F. P. Peterse, Eveline A. M. Heijnsdijk, Nicolien T. van Ravesteyn, Iris Lansdorp-Vogelaar, Inge M. C. M. de Kok

**Affiliations:** Department of Public Health, Erasmus MC, University Medical Center, Rotterdam, The Netherlands

**Keywords:** Health policy, Population screening, Cancer screening, Cancer screening

## Abstract

**Background:**

Many breast, cervical, and colorectal cancer screening programmes were disrupted due to the COVID-19 pandemic. This study aimed to estimate the effects of five restart strategies after the disruption on required screening capacity and cancer burden.

**Methods:**

Microsimulation models simulated five restart strategies for breast, cervical, and colorectal cancer screening. The models estimated required screening capacity, cancer incidence, and cancer-specific mortality after a disruption of 6 months. The restart strategies varied in whether screens were caught up or not and, if so, immediately or delayed, and whether the upper age limit was increased.

**Results:**

The disruption in screening programmes without catch-up of missed screens led to an increase of 2.0, 0.3, and 2.5 cancer deaths per 100 000 individuals in 10 years in breast, cervical, and colorectal cancer, respectively. Immediately catching-up missed screens minimised the impact of the disruption but required a surge in screening capacity. Delaying screening, but still offering all screening rounds gave the best balance between required capacity, incidence, and mortality.

**Conclusions:**

Strategies with the smallest loss in health effects were also the most burdensome for the screening organisations. Which strategy is preferred depends on the organisation and available capacity in a country.

## Background

Many European countries have adopted mass screening programmes for breast, cervical, and colorectal cancer. These screening programmes aim to detect pre-cancerous lesions and early stage cancers to allow for removal of lesions before progression to tumours and treatment of early stage cancers. Due to the early detection and treatment, screening programmes for breast, cervical, and colorectal cancer reduce cancer-specific mortality.^1–3^

The coronavirus disease 2019 (COVID-19) pandemic has affected cancer screening and treatment activities worldwide. In many countries, cancer screening programmes were paused since March 2020 causing a screening disruption for an unknown period of time.^4^ Data from the nationwide Netherlands Cancer Registry showed that the number of breast, gynaecological, and gastrointestinal cancer diagnoses decreased steeply right after the start of the screening disruption.^5^ It is likely that the disruption of screening activities explains, at least partly, this decrease in cancer diagnoses. In addition, many cancer treatments were delayed because of the increased infection risk in hospitals and a reduced hospital capacity for non-COVID patients.^6^ Prior studies have shown that a 6-month delay between a positive screening test and diagnostic testing led to reductions in prevented cervical and colorectal cancers and a less favourable stage distribution for breast and colorectal cancer.^7^ An Australian modelling study estimated that a screening and treatment delay of 6 months would lead to progression from stage I to stage II cancer in 5% of breast cancers and 3% of colorectal cancers (detected and undetected).^8^ These findings suggest that after the screening disruption more diagnoses will be classified as later stage cancer. The delay in screening and treatment of breast and colorectal cancer due to the COVID-19 pandemic was estimated to lead to an increase in cancer-specific deaths of 1% over a period of 10 years in the USA.^9^ However, it can be expected that the organisation of cancer screening highly influences the effect size of a screening disruption. Therefore, it is unknown what the effects of the screening disruption are on cancer incidence and cancer-specific mortality in Europe.

Besides screening organisation, the effects of the screening disruption are expected to be influenced by the length of the disruption and the way screening programmes are restarted after the disruption. Different restart strategies vary in whether screens are delayed or can be caught up, how fast this catch-up will be, whether screens are omitted because of the upper age limit, and which individuals are affected. This information is important for policy makers to decide which restart strategy to implement. Next to effects on incidence and mortality, policy makers are also interested in the screening capacity required per restart strategy to decide whether implementation is possible. At the moment, not much is known about the effects of restart strategies after a screening disruption. Therefore, the aim of this study is to estimate the effects of different restart strategies for breast, cervical, and colorectal screening after the COVID-19 disruption, using microsimulation models.

## Methods

In this study, the effects of a 6-month disruption and different restart strategies were estimated using three MIcrosimulation SCreening ANalysis (MISCAN) models, specified for breast, cervical, and colorectal cancer screening (MISCAN-Breast,^10^ MISCAN-Cervix,^11^ MISCAN-Colon^12,13^). The three MISCAN models were developed by the Erasmus MC and simulate individual life histories of a population and, in a subset, the natural history of breast, cervical, or colorectal cancer, respectively. In addition, screening programmes can be simulated to estimate the effects of screening protocols on required screening capacity, cancer incidence, and cancer-specific mortality. In this study, the models simulated the screening activities using Dutch population and screening data. The models MISCAN-Breast,^10^ MISCAN-Cervix,^11^ and MISCAN-Colon^12,13^ are described in detail elsewhere.

### Dutch national screening programmes

The Dutch breast cancer screening programme entails biennial digital mammography in screening centres and mobile units for women aged 50–75 years.^14^ Because screening mainly takes place in mobile units, appointments are planned based on postal code. Therefore, the actual age at which a woman is screened differs per individual and is somewhere between the exact screening age and the 2 years after that. The mammograms are scored independently by two radiologists according to the Breast Imaging Reporting and Data System (BIRADS) classification. If the two radiologists report different BIRADS classifications, a third radiologist scores the mammogram. Women with BIRADS scores 4, 5, or 0 are referred to an outpatient clinic for additional imaging and possibly a biopsy.

The Dutch cervical cancer screening programme entails cervical swabs at the general practitioner (GP) in women aged 30, 35, 40, 50, and 60 years.^15^ First, the swabs are tested for high-risk human papillomavirus (hrHPV). In case of a positive hrHPV test, the same swab is tested on cytology. Women can also request a self-sampling test on which hrHPV can be tested. In case of a positive self-sampling test, women are advised to go to the GP for a swab that can be tested on cytology. Women with a normal cytology result receive a repeat cytology test after 6 months, whereas women with an abnormal result are directly referred for colposcopy. Women who test hrHPV positive at age 40, 50, or 60 years are invited again at age 45, 55, or 65 years. Also non-attenders at age 40 or 50 years are invited for screening at age 45 or 55 years.

The Dutch colorectal cancer screening programme entails biennial faecal immunochemical test (FIT) for men and women aged 55–75 years.^16^ Individuals testing with a concentration exceeding the cut-off of 47 μg haemoglobin/g faeces are referred for diagnostic colonoscopy. Participants with a negative colonoscopy or colonoscopy with a single small distal tubular adenoma are re-invited in the programme after 10 years.

### Disruption and restart strategies

This study estimated the effects of five restart strategies after a disruption of 6 months (Table 1). In the first strategy (no catch-up), the screening activity during the disruption period was cancelled and not caught up on. The screening activity after the disruption continued as planned. In the second strategy (everyone delay), all screening activity was postponed by the length of the disruption and continued in the order it was planned for the entire population until the stopping age. In breast cancer, this means that the last screen (between age 74 and 75.9 years) was omitted for only a fourth of the individuals (i.e. the women who were planned to be screened between age 75.5 and 75.9 years would be delayed till after the stopping age). In cervical cancer, this means that the additional screen at age 65 years (for women who tested hrHPV positive at age 60 years) was omitted. In colorectal cancer, this means that all screens at age 75 years were omitted for everyone. In the ‘everyone delay’ strategy, the increased interval was not caught up on. The third strategy (first rounds no delay) was similar to the ‘everyone delay’ strategy; however, screening was not delayed for individuals who reach the first screening age after 2020. The fourth strategy (continue after stopping age) was similar to the ‘everyone delay’ strategy; however, the stopping age of the screening protocol was increased by the length of the disruption to ensure the same number of lifetime screening invitations as would have been the case without the disruption. In the last strategy (catch-up after stop), the disrupted screening activity was delayed for the length of the disruption. The screening activity planned after the disruption was not affected. Therefore, catch-up takes place at the same time as regular screening activity. The group of individuals who had one increased screening interval due to the disruption had a decreased interval for the screening round following the delayed round (i.e. an interval of 2.5 years followed by an interval of 1.5 years for breast and colorectal cancer screening and an interval of 5.5 years followed by an interval of 4.5 years for cervical cancer screening). In addition, the stopping age was increased by the duration of the disruption for the individuals who were due for their last screening appointment at the time of the disruption. This was done to ensure that these individuals receive the same number of lifetime screening invitations as would have been the case without the disruption.

**Table 1:** Characteristics of the investigated restart strategies.

Restart strategy	Population affected	Duration of effects	Changes in stopping age
No catch-up	Population due for a screening appointment during the disruption	Only effects during the disruption	No changes in stopping age were needed
Everyone delay	Total population	The delay will exist forever	Individuals exceeding the original stopping age due to the delay missed their last invitation
First rounds no delay	Total population except individuals who reach the first screening age after 2020	All individuals eligible for screening in or before 2020 are delayed for all screening rounds	Individuals exceeding the original stopping age due to the delay missed their last invitation
Continue after stopping age	Total population	The delay will exist forever	The stopping age increased with the duration of the disruption
Catch-up after stop	Population due for a screening appointment during the disruption	The delay is caught up in the second half of 2020	The stopping age increased with the duration of the disruption for the individuals who were invited for their last round in 2020

### Model parameters

In this study, the models simulated a population of 500 million individuals to allow for robust estimates of differences between scenarios. The individuals were at average risk of cancer diagnosis and population characteristics were based on data from Statistics Netherlands^17^ (i.e. birth and life tables) and the Netherlands Comprehensive Cancer Organisation^18^ (cancer incidence and mortality). The screening disruption was modelled for the first 6 months of 2020. The assumption was made that the disruption in screening activity did not influence screening attendance after the disruption. Also, it was assumed that screening capacity was restored to at least 100% directly after the screening disruption.

### Outcomes

Required screening capacity, cancer incidence, and cancer-specific mortality data from the models were transposed to rates per 100,000 individuals (per 100,000 women for breast and cervical cancer) in the total population. Screening capacity was split up into two outcome variables: rate of primary screening tests performed per year compared to undisrupted screening and rate of follow-up tests compared to undisrupted screening. In breast cancer screening, follow-up testing was defined as the number of referrals after a primary screen; in cervical cancer screening, this was defined as the number of colposcopies performed; and in colorectal cancer screening, this was defined as the number of colonoscopies performed. Long-term cancer incidence and cancer-specific mortality rates were compared to model-predicted cancer-specific incidence and mortality rates in a situation with undisrupted screening.

### Sensitivity analyses

Sensitivity analyses were performed to estimate the effects of a disruption of 3, 9, or 12 months for all investigated restart strategies. For the ‘catch-up after stop’ strategy, the catch-up period was assumed to have the same length as the disruption period.

## Results

### Required screening capacity

In the period 2020–2030, the required primary screening capacity for a situation with undisrupted screening was estimated to decrease for breast cancer (11,744–11,080 per 100 000), drop in 2022 for cervical cancer (5439–4116 per 100,000) and subsequently increase (4401 per 100,000), and increase for colorectal cancer (10,128–11,317 per 100,000) (Table 2). The required follow-up test capacity followed similar patterns (Table 3).

**Table 2:** Rate of primary screening tests required per restart strategy after a 6-month disruption compared to undisrupted screening, per 100,000 individuals per year (in %).

	Breast cancer						Cervical cancer						Colorectal cancer					
	Undisrupted screening	No catch-up^a^	Everyone delay^b^	First rounds no delay^c^	Continue after stopping age^d^	Catch-up after stop^e^	Undisrupted screening	No catch-up^a^	Everyone delay^b^	First rounds no delay^c^	Continue after stopping age^d^	Catch-up after stop^e^	Undisrupted screening	No catch-up^a^	Everyone delay^b^	First rounds no delay^c^	Continue after stopping age^d^	Catch-up after stop^e^
2020^f^	11,744	−50%	−51%	−50%	−50%	0%	5409	−50%	−50%	−44%	−50%	0%	10,128	−50%	−53%	−47%	−50%	0%
2021	11,895	0%	−2%	2%	−1%	0%	5439	0%	0%	0%	0%	0%	10,632	0%	−8%	−8%	−3%	0%
2022	11,719	0%	−1%	−1%	0%	0%	4116	0%	18%	17%	18%	0%	10,400	1%	−6%	0%	1%	0%
2023	11,733	0%	−2%	2%	−1%	0%	4100	0%	1%	0%	1%	0%	10,739	0%	−8%	−8%	−2%	0%
2024	11,601	0%	−1%	−2%	0%	1%	4156	0%	0%	0%	0%	0%	10,656	1%	−6%	0%	0%	0%
2025	11,603	0%	−2%	2%	0%	0%	4150	0%	−1%	8%	−1%	0%	10,974	0%	−7%	−7%	−2%	0%
2026	11,382	0%	−1%	−1%	0%	1%	4184	0%	0%	0%	0%	0%	10,973	1%	−5%	0%	0%	0%
2027	11,410	0%	−2%	2%	−1%	0%	4198	0%	0%	0%	0%	0%	11,122	0%	−6%	−7%	−1%	0%
2028	11,227	0%	−1%	−1%	0%	1%	4261	0%	0%	0%	0%	0%	11,313	0%	−8%	−3%	−1%	0%
2029	11,225	0%	−2%	2%	−1%	0%	4343	0%	−1%	−1%	−1%	0%	11,085	0%	−5%	−5%	1%	0%
2030	11,080	0%	−1%	−2%	0%	1%	4401	0%	0%	9%	0%	0%	11,317	0%	−7%	−3%	−2%	0%

^a^The screening activity during the disruption period was cancelled and not caught up on.

^b^All screening activity was postponed by the length of the disruption.

^c^All screening activity was postponed by the length of the disruption, but not for individuals who reached the first screening age after 2020.

^d^All screening activity was postponed by the length of the disruption and the stopping age is increased by the length of the disruption.

^e^The disrupted screening activity was caught up immediately after the disruption at the same time as regular screening activity.

^f^Because of the disruption, all screens in 2020 were performed in the second half of the year. Therefore, a capacity change of −50% in 2020 represented a normal screening capacity during the second half of 2020 and a capacity change of 0% in 2020 represented a double capacity during the second half of 2020.

**Table 3:** Rate of follow-up tests required per restart strategy after a 6-month disruption compared to undisrupted screening, per 100,000 individuals per year (in %).

	Breast cancer						Cervical cancer						Colorectal cancer					
	Undisrupted screening	No catch-up^a^	Everyone delay^b^	First rounds no delay^c^	Continue after stopping age^d^	Catch-up after stop^e^	Undisrupted screening	No catch-up^a^	Everyone delay^b^	First rounds no delay^c^	Continue after stopping age^d^	Catch-up after stop^e^	Undisrupted screening	No catch-up^a^	Everyone delay^b^	First rounds no delay^c^	Continue after stopping age^d^	Catch-up after stop^e^
2020^f^	247	−50%	−45%	−44%	−44%	8%	196	−50%	−51%	−42%	−51%	−13%	447	−35%	−36%	−32%	−34%	1%
2021	251	0%	10%	13%	12%	0%	201	0%	−3%	1%	−3%	12%	465	0%	−4%	−4%	1%	0%
2022	248	19%	6%	6%	8%	−6%	163	0%	13%	14%	13%	0%	463	7%	−2%	1%	3%	−1%
2023	250	0%	1%	3%	2%	−1%	160	0%	−2%	−1%	−2%	0%	479	−5%	−10%	−9%	−5%	0%
2024	248	3%	0%	0%	2%	0%	162	0%	−3%	−1%	−3%	0%	484	2%	−3%	0%	2%	0%
2025	248	0%	0%	2%	2%	0%	164	0%	−5%	5%	−5%	−1%	495	−2%	−8%	−7%	−3%	0%
2026	245	1%	0%	−1%	2%	0%	166	0%	−4%	1%	−3%	0%	506	−1%	−4%	−2%	−1%	0%
2027	246	0%	−1%	2%	1%	0%	167	0%	−4%	−1%	−3%	0%	520	2%	−3%	−3%	1%	0%
2028	243	1%	0%	−1%	2%	1%	170	0%	−4%	−2%	−4%	0%	526	−1%	−6%	−4%	−1%	0%
2029	243	0%	−1%	2%	1%	0%	172	0%	−4%	−2%	−3%	0%	528	0%	−3%	−2%	1%	0%
2030	242	0%	0%	−1%	1%	1%	175	1%	−4%	5%	−3%	0%	538	−1%	−6%	−3%	−2%	0%

^a^The screening activity during the disruption period was cancelled and not caught up on.

^b^All screening activity was postponed by the length of the disruption.

^c^All screening activity was postponed by the length of the disruption, but not for individuals who reached the first screening age after 2020.

^d^All screening activity was postponed by the length of the disruption and the stopping age is increased by the length of the disruption.

^e^The disrupted screening activity was caught up immediately after the disruption at the same time as regular screening activity.

^f^Because of the disruption, all screens in 2020 were performed in the second half of the year. Therefore, a capacity change of −50% in 2020 represented a normal screening capacity during the second half of 2020 and a capacity change of 0% in 2020 represented a double capacity during the second half of 2020.

For all cancer sites, the ‘catch-up after stop’ strategy required a yearly primary screen capacity equal to a situation with undisrupted screening. However, in 2020, all screening activity took place in the second half of the year. Therefore, the required capacity during the second half of 2020 was actually doubled in the ‘catch-up after stop’ strategy. The strategies ‘no catch-up’, ‘everyone delay’, ‘first rounds no delay’, and ‘continue after stopping age’ required a reduced capacity in 2020, followed by an equal or slightly reduced capacity in the years after the disruption. In 2022, the year of the second round in the new Dutch cervical cancer screening programme, the ‘everyone delay’, ‘first rounds no delay’, and ‘continue after stopping age’ strategies required an additional capacity of 17–18% compared to undisrupted screening.

The effects of the restart strategies on the required follow-up test capacity were similar to the effects on the required primary screening test capacity. Moreover, the ‘catch-up after stop’ strategy will require an increased follow-up capacity compared to undisrupted screening in 2020 for breast cancer and colorectal cancer (8 and 1%, respectively) leading to a more than doubled required follow-up capacity, because all screening took place in the second half of 2020. For cervical cancer, the required follow-up capacity for the ‘catch-up after stop’ strategy was −13% in 2020, which comes down to a 75% increase in the second half of the year, when all screening took place. Next to that, the required cervical cancer follow-up capacity remained increased in 2021 (12%). Furthermore, the required follow-up capacity for breast cancer screening in the ‘everyone delay’, ‘first rounds no delay’, and ‘continue after stopping age’ strategies were increased in 2021 and 2022. Additionally, the ‘no catch-up’ strategy required an increased follow-up capacity in breast and colorectal cancer screening in the year of the next screening round for individuals who missed their screen due to the disruption.

### Cancer incidence

**Fig. 1:**
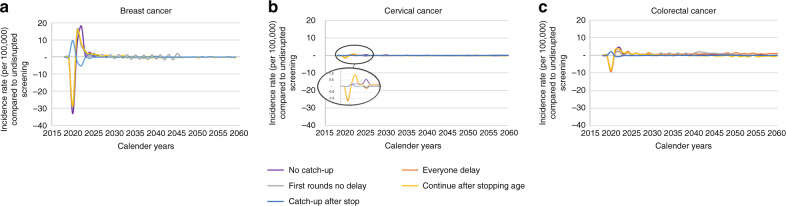
Cancer incidence rate (per 100,000) after a 6-month disruption compared to undisrupted screening over time for the different restart strategies. **a** Breast cancer, **b** Cervical cancer, **c** Colorectal cancer.

In breast and colorectal cancer, the ‘catch-up after stop’ strategy was estimated to lead to an increased incidence rate compared to undisrupted screening in 2020, followed by a small decrease in incidence in the year of the next screening appointment for the population which was disrupted (Fig. 1). On the contrary, the other four strategies were estimated to lead to an incidence drop in 2020, followed by an increased incidence for 2 years. This drop was larger for breast cancer (−29 per 100,000) than for colorectal cancer (−9 per 100,000). After 2025, all restart strategies had only minor deviations in incidence rate compared to undisrupted screening. For cervical cancer, all restart strategies resulted in similar patterns as for breast and colorectal cancer, though the effect size was much smaller and some increases in incidence occurred a year later.

### Cancer-specific mortality

**Fig. 2:**
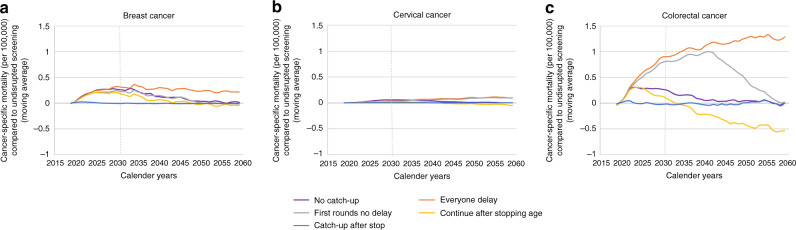
Moving average of cancer-specific death rate (per 100,000) after a 6-month disruption compared to undisrupted screening over time for the different restart strategies. **a** Breast cancer, **b** Cervical cancer, **c** Colorectal cancer. The vertical dotted line represents the cut-off used in Fig. 3.

In Fig. 2, the cancer-specific mortality rates compared to undisrupted screening are shown as a moving average over 3 years per cancer site. The ‘catch-up after stop’ strategy resulted in a cancer-specific mortality rate similar to that for undisrupted screening between 2020 and 2060 in the three cancer sites. On the contrary, the ‘everyone delay’ strategy led to the largest increase in cancer-specific mortality rate over time (0.4 per 100,000 in breast cancer, 0.1 per 100,000 in cervical cancer, and 1.4 per 100,000 in colorectal cancer).

In the first years after disruption, the ‘no catch-up’, ‘first rounds no delay’, and ‘continue after stopping age’ strategies resulted in similar cancer-specific mortality rates as the ‘everyone delay’ strategy. After 2023, 2059, and 2040, the ‘no catch-up’ and ‘first rounds no delay’ strategies led to decreasing mortality rates for breast, cervical, and colorectal cancer, respectively. For the ‘no catch-up’ strategy, the mortality rates returned to be equal to undisrupted screening after 2048, 2058, and 2056. For the ‘first rounds no delay’ strategy, the mortality rates returned to be equal to undisrupted screening after 2050, 2085, and 2057. After 2023, 2031, and 2022, the ‘continue after stopping age’ strategy led to decreasing mortality rates for the three cancer sites, respectively. These mortality rates returned to be equal to undisrupted screening after 2047, 2040, and 2034.

**Fig. 3:**
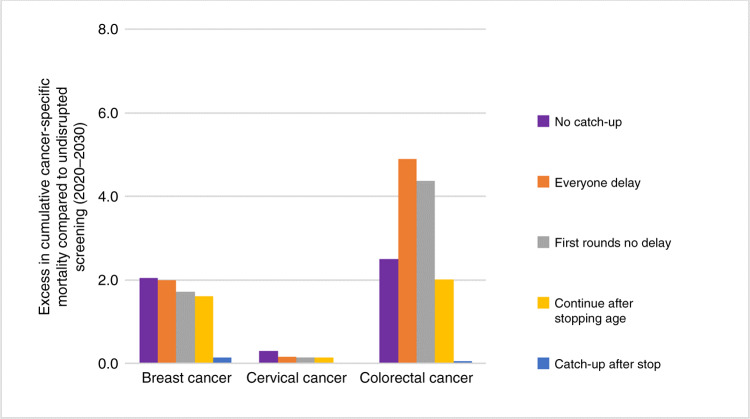
Cumulative excess in cancer-specific mortality rate (per 100,000) after a 6-month disruption compared to undisrupted screening over the years 2020–2030 for the different restart strategies.

The cumulative breast cancer and cervical cancer mortality rates over the 10 years following the screening disruption (2020–2030) were the highest in the ‘no catch-up’ strategy (Fig. 3). The cumulative mortality rate was 2.0 per 100,000 for breast cancer (186 cases in the Dutch situation) and 0.3 per 100,000 for cervical cancer (27 cases in the Dutch situation). In colorectal cancer, the ‘everyone delay’ strategy led to the highest cumulative mortality rate (4.9 per 100,000; 740 cases in the Dutch situation). Smaller cumulative mortality rates were found for the other restart strategies, with the smallest rates for the ‘catch-up after stop’ strategy in all cancer sites. The absolute differences in cumulative cervical cancer mortality rates between the five restart strategies were small. In breast and cervical cancer, the ‘no catch-up’ strategy led to the highest mortality rates, whereas in colorectal cancer, the ‘everyone delay’ and ‘first rounds no delay’ strategies resulted in higher mortality rates.

### Sensitivity analysis

In general, a delay of 3 months led to a lower cancer-specific mortality, while the 9- and 12-month delays resulted in higher cancer-specific mortalities than for a 6-month delay (Supplement Fig. 1). Relative differences between the restart strategies and cancer sites remained the same. The relative differences in mortality between disruptions of 3, 6, 9, or 12 months were the largest in breast cancer.

## Discussion

Using well-validated microsimulation models for three cancer sites, this study found that the impacts of a screening disruption for breast and colorectal cancer are substantial. For cervical cancer, the disruption had less influence. Furthermore, we showed that the size of the burden will be influenced by the restart strategy, whereby catching up on the missed screening activity would have the smallest effects on incidence and mortality, but the biggest effect on screening capacity. The other investigated restart strategies required a screening capacity similar to undisrupted screening. Among these, the cancer incidence and cancer-specific mortality were most favourable when screening was continued after the stopping age to allow for a similar number of screening rounds for the target population as without disruption.

The overall patterns in effects of the restart strategies were similar for the three cancer sites, but the effect sizes were different. The effects on incidence were the largest for breast cancer, smaller for colorectal cancer, and minimal for cervical cancer. These differences in effect size are caused by the difference in absolute cancer incidence, screening interval, and/or dwelling time between the cancer sites. In case of a shorter interval between screen tests, the relative increase in waiting time for the next round due to a 6-month disruption is larger. Because of a relative lower incidence, longer screening interval, and larger dwelling time, the effects of the disruption and the restart strategies on cervical cancer incidence were small. It was remarkable that the cancer-specific mortality in colorectal cancer was much higher in the strategies in which the stopping age was not increased (‘everyone delay’ and ‘first rounds no delay’) than in the strategies that did increase the stopping age (‘continue after stopping age’ and ‘catch-up after stop’). These differences can be explained due to the fact that in colorectal cancer all delayed individuals missed their last screening round in the ‘everyone delay’ and ‘first rounds no delay’ strategy. In case of breast cancer screening, due to a disruption of 6 months out of an interval of 24 months, one out of four individuals missed their last screening round (since we assumed screening appointments to be planned based on postal code instead of date of birth). In case of cervical cancer screening, only the additional screen at age 65 years was omitted, which was only offered to women who tested hrHPV positive at age 60 years. Therefore, the difference between the ‘everyone delay’ and ‘continue after stopping age’ strategies is bigger for colorectal than for breast and cervical cancer screening.

Nation-wide organised cancer screening programmes are known to reduce inequality between individuals with different socio-economic status.^19^ To maintain this after a screening disruption, it is important that the restart of screening activity is well organised. The feasibility of the four restart strategies depends on the capacity available and the way screening programmes are set up in a country or region. In 2017, 68% of European countries indicated a limited capacity of the screening programme.^20^ The limitations differed from a shortage of screening personnel to limitations in screening materials, laboratory capacity, follow-up tests, and insufficient financial resources. In the Netherlands, the breast cancer screening capacity is limited for primary screens due to a shortage of screening unit personnel, whereas the colorectal cancer screening capacity is limited by the colonoscopy capacity.^16,21^ The specific limitations determine whether a country or region is able to reach the required capacity for the investigated restart strategies. Furthermore, practical issues can arise based on the way a screening programme is set up. For example, a programme with a fixed number of mobile breast cancer screening units is not able to catch-up disrupted screening and continue the originally scheduled screens at the same time for two different locations. Also, cervical cancer screening programmes can have limitations in analysing the hrHPV samples, because the laboratory equipment might be used for COVID-19 testing.

The results in this study were based on the screening situation in the Netherlands. In absolute numbers (based on the increase in incidence rate), the results estimated 145 additional breast cancer deaths, 13 additional cervical cancer deaths, and 307 additional colorectal cancer deaths between 2020 and 2030 for the ‘continue after stopping age’ strategy compared to undisrupted screening. Despite the additional deaths compared to a situation without screening disruption, the screening programmes were estimated to still prevent 12,537 breast cancer, 2655 cervical cancer, and 14,190 colorectal cancer deaths in this period in the Netherlands. This study did not investigate the effects of the disruption and the restart strategies on the amount of overdiagnosis. However, we expect that overdiagnosis will be lower for the first screening round after the disruption due to the increased screening interval. Furthermore, we expect that overdiagnosis can increase in the restart strategies that increase the stopping age, but we expect this increase to be small, because the stopping age was only increased by 6 months.

We expect that the capacity, incidence, and mortality rates can be applied to other countries or regions with comparable screening strategies. For countries with significant differences in screening programmes compared to the Dutch programme, the effects of the disruption and the restart strategies can differ. For example, an upper age limit of 69 years for breast cancer screening instead of 75 years leads to a smaller population eligible for screening. Therefore, a smaller population is affected by the screening disruption, leading to smaller effect sizes. Next to that, for an annual screening interval in colorectal cancer screening instead of a biennial interval, the disruption becomes proportionately larger, which can lead to larger effect sizes. Also, the use of a different screening test can influence whether the effects found are applicable to other countries. For example, the use of a cytology test only in cervical cancer screening instead of a combination of hrHPV and cytology tests can lead to different effect sizes. Especially in countries with opportunistic screening, the effects of a screening disruption are expected to differ a lot. Next to that, differences in results may be expected for countries or regions with a different population composition or a different population risk to develop breast, cervical, or colorectal cancer.

In practice, the Dutch breast cancer screening was disrupted for 3 months, cervical cancer screening for 3.5 months, and colorectal cancer for 2 months. Sensitivity analyses showed that a screening disruption of 3 months led to smaller effect on capacity, incidence, and mortality. However, the programmes were not able to restart at full capacity due to hygiene and safety restrictions. Therefore, a part of the population will have a longer screening delay than the duration of the disruption. In the case of a 3-month disruption followed by 6 months with 50% capacity, nearly all screens will be delayed for 6 months, which implies that the effects are comparable to a 6-month disruption followed by full capacity.

An important strength of this analysis is the timely response to the current screening situation and the use of well-validated models. However, this study also has some limitations. In the models, it was assumed that attendance to the screening programmes was equal to the attendance rates before the screening disruption. In case attendance rates decrease after the disruption, we expect required capacity to be lower and cancer-specific mortality to be higher. Also, it was assumed that the screening programmes did not face further hygiene or safety restrictions because of the COVID-19 pandemic as soon as the screening disruption was over. In case of additional hygiene and safety restrictions after the disruption, the available capacity is expected to be low. This low capacity can lead to longer delays in screening for part of the population resulting in higher cancer-specific mortality rates. Furthermore, the assumption was made that other cause mortality did not change due to the COVID-19 pandemic, although it can be expected that it has an effect on mortality, especially in older age groups. We expect that cancer-specific mortality rates will be slightly lower if a higher other cause mortality is taken into account.

Notwithstanding these limitations, this study provides an important first peek in the potential impact of the screening disruptions on resource requirements and long-term benefits of existing screening programmes. It underlines the importance of careful consideration of the restart strategy to mitigate the negative impact of these disruptions. At the moment, this has become an important topic, because many countries were strained to disrupt their screening programmes due to the COVID-19 pandemic. This study provides well-grounded estimates on the requirements and effects of screening restart strategies for policy makers of national or regional cancer screening organisation so that they can make informed decisions on how to restart their screening programmes.

In conclusion, this study found that catching up on the delayed screening activity would result in the smallest effects on cancer incidence and cancer-specific mortality. However, this restart strategy requires a very high screening capacity in a short time period. A restart strategy in which all screening is delayed and the stopping age is increased requires a screening capacity similar to a situation without screening disruption and results in minimal effects on incidence and mortality.

## Supplementary information

Supplemental figure

## Data Availability

Detailed modelling results are available upon request.
