# Supplementary material for: Effects of cancer screening restart strategies after COVID-19 disruption
Source: Br J Cancer. 2021 Mar 15;124(9):1516–23. doi: 10.1038/s41416-021-01261-9 (PMC7957464; doi:10.1038/s41416-021-01261-9)

Effects of Cancer Screening Restart Strategies after COVID-19 Disruption - Supplement

Supplement figure 1. Excess in cumulative cancer-specific mortality rate (per 100 000) between 2020 and 2030 for the different restart strategies after disruptions of 3, 6, 9, and 12 months. A) Breast cancer, B) Cervical cancer, C) Colorectal cancer

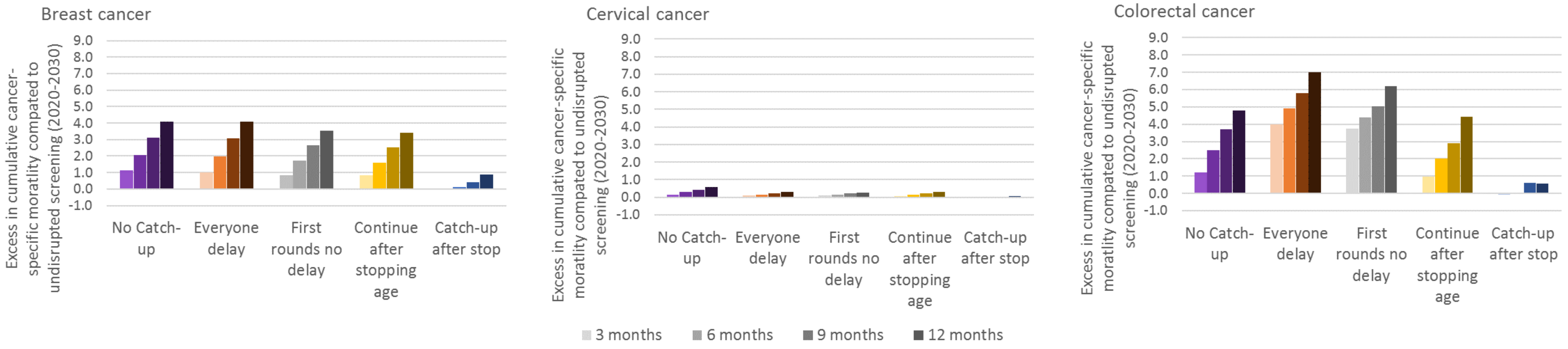

Supplement: Supplementary file 1 — Supplemental figure [file 41416_2021_1261_MOESM1_ESM.pdf]
